# Long-term follow-up of retropupillary iris-claw intraocular lens implantation: a retrospective analysis

**DOI:** 10.1186/s12886-015-0146-4

**Published:** 2015-10-27

**Authors:** Matteo Forlini, Wael Soliman, Adriana Bratu, Paolo Rossini, Gian Maria Cavallini, Cesare Forlini

**Affiliations:** Department of Ophthalmology, Policlinico di Modena, Modena, Italy; Department of Ophthalmology, Assiut University Hospitals, Assiut, Egypt; Department of Ophthalmology, Hospital S. Maria delle Croci, Ravenna, Italy

**Keywords:** Iris-claw, Retropupillary, Sutureless vitrectomy

## Abstract

**Background:**

The ideal intraocular lens in cases of aphakia without capsular support is debated. Choices include anterior chamber lenses, iris- or scleral-sutured lenses, and iris-claw lenses. Our aim was to report our long-term evaluation of the use of retropupillary implantation of the Artisan iris-claw intraocular lens (RPICIOL) in several aphakic conditions without capsular support.

**Methods:**

A retrospective analysis of consecutive 320 eyes of 320 patients (222 males and 98 females) without capsular support in which we performed RPICIOL implantation in post-traumatic aphakia (141 eyes, group 1), post-cataract surgery aphakia (122 eyes, group 2), and in cases in which penetrating keratoplasty was associated with vitrectomy (57 eyes, group 3). Either anterior or posterior vitrectomy procedures were performed with 20–, 23-, or 25-gauge techniques for different associated anterior or posterior segment indications. We reviewed the refractive outcome, anatomical outcome, long-term stability of the implants, and possible long-term complications.

**Results:**

The mean patient age was 59.7 years (range, 16–84 years) in group 1; 60.1 years (range, 14–76 years) in group 2; and 65.8 years (range, 25–71.5 years) in group 3. The mean follow-up time was 5.3 years (range, 1 month to 8 years). At the end of the follow-up period, the mean post-operative best-corrected LogMAR visual acuity was 0.6 (range, perception of light to 0.3) in group 1; 0.3 (range, 0.5–0.1) in group 2; and 0.6 (range, hand movement to 0.2) in group 3. Disenclavation of RPICIOLs occurred in three cases because of slippage of one of the iris-claw haptics and spontaneous complete posterior dislocation occurred in one case. One case presented with retinal detachment, and no cases of uveitis were observed. Eight cases complained of chronic dull pain, and severe iridodonesis was seen in five cases. One case of post-operative macular edema was observed without post-operative increase in the mean intraocular pressure. There was no statistically different change in the endothelial cell density (cells/mm^2^) at the end of the follow-up period.

**Conclusions:**

RPICIOL for secondary implantations is a valid alternative strategy to scleral-fixated or angle-supported IOL implantation.

## Background

The ideal intraocular lens in cases of aphakia without capsular support is debated. Choices include anterior chamber lenses, iris- or scleral-sutured lenses, and iris-claw lenses, with the latter now being used more commonly. The retropupillary approach for iris-claw intraocular lens (RPICIOL) implantation has recently gained popularity. In 1971, Worst first presented the iris-claw lens (a biconvex polymethyl methacrylate IOL fixated above the iridal plane at the mid-periphery of the iris) at a meeting in Paris [1]. Although Amar [2] published the retropupillary implantation technique using an iris-claw IOL as early as 1980, and Rijneveld et al. [3] reported clinical results in 1994, it was only after the new description by Mohr et al. in 2002 [4] that this approach gained popularity. More recently, a new issue has been added to the debate regarding the best choice of IOL for correcting aphakia: where to position the iris-claw lens inside the eye. Some studies recommend positioning the iris-claw lenses above the iris in cases of aphakia [5–9], while others recommend a retropupillary position [3, 4, 10–14].

The aim of this retrospective study was to evaluate the feasibility and safety of retropupillary implantation of the Artisan iris-claw lens in different aphakic situations without adequate capsular support.

## Methods

This was a retrospective study of RPICIOL implantation in 320 eyes of 320 patients (222 males and 98 females) at the Department of Ophthalmology, Hospital S. Maria delle Croci, Ravenna, Italy from January 2002 to December 2009. All patients gave signed informed consent following a discussion of the details of the intervention and the possible risks. The study followed the guidelines of the Declaration of Helsinki and the study protocol was approved by the committee of medical ethics of the Hospital S. Maria delle Croci, Ravenna, Italy.

### Inclusion criteria

Group 1: Subjects suffered post-traumatic subluxation or total dislocation of the crystalline lens or ruptured cataractous lens without adequate capsular support, with or without severe lacerations of the iris that required reconstruction. Cases with or without traumatic retinal detachment were included. Aphakia in these cases resulted from the severe intraocular trauma and patients required both anterior and posterior segment reconstruction. All RPICIOL implantations were primary procedures during the main reconstructive procedures.

Group 2: Subjects suffered post-cataract surgery aphakia that resulted from intra- or post-operative complications, which included dropped fragments or the whole nucleus of the crystalline lens. Cases with subluxation or total dislocation of the IOL that required both anterior and posterior vitrectomy were also included. RPICIOL implantations were primary procedures during intra-operative complications and secondary procedures following post-operative complications. All were one-step procedures.

Group 3: Subjects included cases in which keratoplasty was performed in association with vitrectomy. In these cases, we used a Landers temporary keratoprosthesis (Ocular Instruments Inc., Bellevue, WA, USA) during vitrectomy before completion of the keratoplasty procedure.

### Exclusion criteria

Were rubeosis iridis and total aniridia. Pre-operatively, we performed the following investigations: best-corrected visual acuity using a LogMAR chart, keratometry, biometry, and anterior segment examination with slit lamp. Post-operatively, we examined patients on the 1^st^, 7^th^, and 30^th^ post-operative days, and then annually in the follow-up period. At post-operative follow-up assessments, we assessed best-corrected visual acuity using LogMAR, IOP, and anterior segment examination with slit lamp. Pre-operatively, endothelial cell density (cells/mm^2^) using a non-contact specular microscope (Konan NonCon Specular microscope V, SP-9000; Konan Medical Inc., Hyogo, Japan), was recorded for all patients in group 1 and group 2, and yearly postoperative endothelial cell density (cells/mm^2^) was assessed for the same group. Endothelial cell density was also assessed in group 3, but with pre-operative eye bank assessment.

### Surgical technique

The lens used in this study was the Artisan aphakia IOL (Ophtec BV, Groningen, The Netherlands) which is a polymethyl methacrylate IOL with an 8.5-mm length, 1.04-mm maximum height, and 5.4-mm optical zone width. The optic power was calculated using the SRK/T formula with the aim of achieving emmetropia. The manufacturer’s recommendation for A constant is 115.0 for implantation above the iris but we used an A constant of 116.5 because we implanted the lens retropupillary.

All surgeries were performed by two experienced surgeons under either general or peribulbar anesthesia and both surgeons used the same surgical technique. One corneal paracentesis was performed at the side of the surgeon’s non-dominant hand at either the 3 or 9 o’clock position. Acetylcholine 1 % was injected intracamerally through the paracentesis for miosis, followed by injection of a dispersive cohesive viscoelastic material (IAL; Bausch & Lomb, Rochester, NY, USA). A 5.4-mm corneal incision was made at 12 o’clock and the iris-claw IOL was then inserted upside down (with its convex surface placed posteriorly), rotated with an Artisan lens forceps to a horizontal position, and centered on the pupil. The optic of the reversed iris-claw IOL was held securely with forceps. Next, the two haptics were gently slid behind the iris and the optic was lifted slightly forward toward the posterior surface of the iris so that the claw configuration of the haptic could be recognized from above on the iris anterior surface. With the other hand, a long micro-spatula was used through a lateral paracentesis at either 3 or 9 o’clock based on the surgeon’s non-dominant hand, to insert iris tissue into the claw. The second haptic was fixated in the same way, using the same spatula. Care was taken to apply gentle pressure over the slotted center of the lens haptic to enclavate a fair amount of iris tissue to avoid ovalisation of the pupil and to decrease the effect of enclavation on the movement of the pupil. We did not perform peripheral iridectomies in any of the cases. At the end of the procedure, we closed the corneal incision with three simple interrupted 10-0 nylon sutures. In cases that were combined with keratoplasty, and after vitrectomy for posterior segment complications using Landers keratoprosthesis, we implanted the lens using an open-sky technique.

We used ophthalmic endoscopy using a fused fiber 30 000-pixel 18G probe containing both the light source and the video input (EndoGnost LS 200, Schwind, Kleinostheim, Germany) to evaluate the state of the remaining capsule and to confirm removal of the entire remnant of the capsule to avoid post-operative fibrosis that could affect the stability of the RPICIOL. We also used endoscopy to evaluate and confirm enclavation of an acceptable amount of iris tissue between the iris-claw, and the strength of fixation of the RPICIOL to the posterior surface of the iris.

## Results

**Fig. 1:**
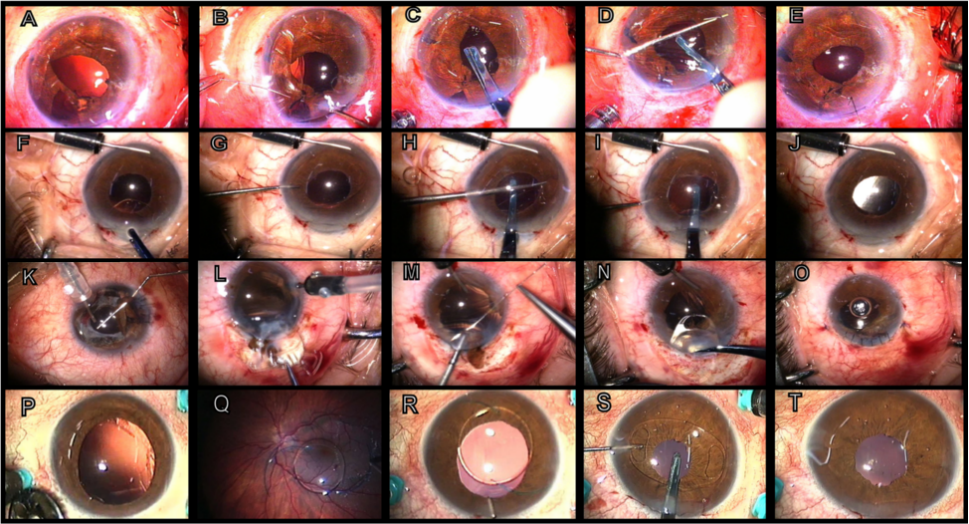
**a**–**e**: RPICIOL implantation in a post-traumatic aphakia case with iridodialysis. **a** Aphakia with large iridodialysis; **b** Iris suturing with 10–0 polypropylene; **c** The IOL was introduced into the anterior chamber and oriented orthogonal to the corneal tunnel; **d** Positioning of the iris-claw lens behind the iris and enclavation of the iris tissue in the right haptic using a long spatula. Next, fixation of the haptic proximal to the paracentesis was performed using the same spatula; **e** Iris reconstruction completed and RPICIOL implanted. **f**–**j**: RPICIOL implantation in a post-cataract surgery aphakia case. **f** 25-gauge infusion into the anterior chamber and introduction of the RPICIOL; **g** The iris-claw lens was rotated and oriented in the horizontal meridian (3 to 9 o’clock) with a hook; **h**–**i** The RPICIOL was held with special forceps and enclavation of iris tissue in the claws of the lens was performed with a long spatula introduced through a lateral paracentesis by applying light pressure on the iris mid-periphery at the site exactly overlying the haptics, first at 9 o’clock then at 3 o’clock; **j** Immediate post-operative outcome; the IOL was stable and centered. **k**–**o**: RPICIOL implantation in a post-traumatic aphakia case. **k** Anterior dislocation of a posterior chamber IOL in the anterior chamber with iris laceration; **l** Removal of the dislocated IOL; **m** Iridoplasty with polypropylene 10–0 suture; **n** Introduction of the iris-claw lens; **o** The eye after finishing the operation. **p**–**t**: RPICIOL implantation in a post-cataract surgery aphakia case. **p**–**q** Posterior dislocation of a posterior chamber IOL in the vitreous cavity; **r** Removal of the posteriorly dislocated IOL after vitrectomy, to the anterior chamber; **s**–**t** Introduction of the iris-claw lens, and the eye after finishing the operation

We included 320 eyes of 320 patients and all 320 eyes underwent RPICIOL implantation. Group A included 141 eyes, group B included 122 eyes (primary implantations following intra-operative complications in 76 cases and secondary implantations following post-operative complications in 46 cases), and group 3 included 57 eyes. The mean baseline best-corrected LogMAR visual acuity was 1.0 (range, from perception of light to 0.5) in group 1, 0.7 (range, 1.0–0.3) in group 2 and 0.8 (range, from hand movement to 0.4) in group 3. The mean patient age was 59.7 years (range, 16–84 years) in group 1, 60.1 years (range, 14–76 years) in group 2, and 65.8 years (range, 25–71.5 years) in group 3. The mean follow-up time was 5.3 years (range, 1 month to 8 years). The post-operative residual spherical equivalent error was−1.42 diopters (D) ± 1.22 (D) standard deviation (SD) in group 1, −1.5 ± 1.15 (D) in group 2, and −2.4 ± 2.1 (D) in group 3. At the end of the follow-up period, the mean post-operative best-corrected LogMAR visual acuity was 0.6 (range, from perception of light to 0.3) in group 1, 0.3 (range, 0.5–0.1) in group 2, and 0.6 (range, from hand movement to 0.2) in group 3. In all cases, the RPICIOLs were in stable position except for three cases that presented with subluxation because of slippage of one of the iris-claw haptics after a mean follow-up of 12 months (range, 10–14 months). RIPICIOLs were refixated in these three cases. Another case presented with complete spontaneous dislocation of the Artisan lens in the vitreous cavity. This case had post-traumatic aphakia, retinal detachment, and an opaque cornea, which were treated by keratoplasty, 23-gauge vitrectomy, and heavy silicone. After 2 months, spontaneous dislocation occurred after removing the silicone. In this case, the Artisan lens explanted and scleral fixation was performed to avoid the stiff, traumatized iris. One case developed retinal detachment after RPICIOL implantation for post-traumatic aphakia. We saw no cases of uveitis after RPICIOL implantation. Eight cases complained of chronic dull eye pain following RPICIOL implantation, which decreased with time. Three cases complained of blurred vision when leaning forward and five cases showed severe iridodonesis. The mean post-operative intraocular pressure in the three groups was 16.4 ± 3.4 mmHg. Three cases in group 1, two cases in group 2, and two cases in group 3 presented with post-operative increased intraocular pressure (24 ± 4.2 mmHg), which was managed medically with topical anti-glaucoma drugs. We had three cases of cystoid macular edema, verified by Stratus optical coherence tomography, which developed 2 months after RPICIOL implantation and vitrectomy for posterior dislocated IOL. Oval pupil was seen in 5 % (16 eyes) of the patients, especially in association with iridoplasty (11 patients). Pigment dispersion was detected in three eyes in group 1. When 20-gauge vitrectomy was a common procedure, we implanted 16 RPICIOLs in 2 years (2002–2003). When we changed to 23-gauge and 25-gauge vitrectomy, we implanted 304 RPICIOLs in 6 years (2004–2009) (Fig. 1).

The pre-operative mean and SD of the endothelial cell density (cells/mm^2^) was (2227.4 ± 524.4 SD) and the mean post-operative values were (2170.4 ± 431.7 SD). This difference was not statistically significant (*P* = 0.96).

## Discussion

Surgical correction of aphakia without capsular support remains a challenge. Each of the available options has its own risks and complications: transscleral fixation of posterior chamber IOLs is an extremely technically demanding procedure with relatively high risk of intra-operative and post-operative complications and requires a large amount of dissection into the conjunctiva and the sclera [15, 16]. Angle-supported anterior chamber IOL implantation, although technically easier, has been associated with several complications related to the iridocorneal angle and the corneal endothelium [17]. Artisan aphakic lenses have been successfully implanted in the anterior chamber and fixated to the anterior surface of the iris in aphakic eyes that had undergone vitrectomy [5, 18, 19]. Retropupillary implantation of the Artisan iris-claw lens after vitrectomy has also been reported [4, 10]. Although implanting the iris-claw lens above the iris for aphakic eyes decreases the endothelial cell count [5, 13, 18, 20–22], in most studies using the retropupillary fixation technique, a significant effect on the endothelial cell count was not found, as in our study [11–14, 23]. Gicquel et al. showed a significant difference in the mean endothelial cell loss between 6 months and 1 year after penetrating keratoplasty and iris-claw IOL implantation, comparing a group of eyes that underwent implantation of iris-claw lenses in the anterior chamber (on the iris) and another group that underwent retropupillary implantation (19 % versus 3.7 %). Although the sample size was small (27 eyes) and the power of the study limited, this difference appeared to favor retropupillary implantation compared with fixation on the front of the iris regarding changes to the corneal endothelium [13].

Two studies of RPICIOL implantation showed pigment dispersion as a complication, but this was not seen in several additional studies [3, 10–12, 14]. We detected pigment dispersion in three of our patients, and we believe that the vaulted design of the Artisan aphakic lens and its inverted position provided adequate space between the iris pigmented epithelium and the optical zone of the lens. This could explain the low number of iris dispersion cases in our study.

Although some authors consider that the possibility of total luxation of the RPICIOL to be remote, an intra-operative case has been reported [24], and we encountered this complication in one eye, which occurred spontaneously 2 months after surgery. We believe that the previously traumatized iris was rigid in this case and did not allow an adequate amount of tissue to be included in the fissures of the claw-like haptics. Therefore, we agree that although this complication is rare, it is definitely possible [12]. Three cases of spontaneous disenclavation of one haptic occurred in our series, a complication that has been reported previously [10, 11, 13].

Some believe it is preferable to perform an IOL exchange rather than a new fixation of the same IOL, particularly in cases of traumatically dislocated iris-claw IOLs, because of the considerable alterations on the affected haptics of the IOLs, which might not guarantee a reliable re-enclavation. However, we easily managed our three cases of subluxation by refixation, with good results [25]. We experienced one case of macular edema, and this has also been reported previously [4, 11]. We believe this was a result of the primary cause of the aphakia or the vitrectomy operation itself.

Ovalisation of the pupil was seen in 5 % of patients and especially in patients who underwent iris reconstruction, as previously reported [13]. In our opinion, this was not an important complication compared with the severity of the initial condition. We encountered no uveitis cases in our patients, consistent with other RPICIOL studies [4, 10–12, 14].

Rijneveld et al. [3] found iridal synechiae in 5 % of patients undergoing RPICIOL implantation and 11 % in patients with implantation above the iris. Gicquel et al. [13] reported iridal synechiae in three of 41 patients with RPICIOL. Iridal synechiae could have resulted from the initial disease in these cases, as both studies involved RPICIOL implantation for patients with aphakia who required keratoplasty because of bullous kertaopathy.

We saw elevated IOP in seven cases and all were managed medically; our results were consistent with previous reports [10, 11]. We did not perform peripheral iridectomies and no cases of pupillary block occurred. This could be explained by the posterior vaulting of this lens when implanted in a reverse position on the back of the iris and the adequate space between the lens optic and the back of the iris.

One case of retinal detachment was reported in our series in a post-traumatic aphakia patient who underwent both RPICIOL and posterior vitrectomy. The retinal detachment could have resulted from the initial disease or the second intervention.

Eight cases complained of dull aching eye pain, three cases had blurred vision when leaning forward, and five cases showed severe iridodonesis. We believe that these effects resulted from the weight of the lens itself pushing the iris toward the cornea when patients leaned forward. The same mechanism could cause chronic dull pain.

When we shifted to 23-gauge and 25-gauge vitrectomy, RPICIOL implantations for managing aphakia without capsular support increased dramatically in our institution. The high feasibility of the technique allowed us to perform new transconjunctival vitrectomy techniques without the need for invasive techniques related to secondary implantation for aphakia without capsular support.

The strengths of this study are the long follow-up and the large number of patients. The limitations of this study are the retrospective design and the lack of comparison with anterior implantation of the iris-claw lens.

## Conclusions

The complications related to RPICIOL implantation were minimal compared with its benefits. Therefore, using retropupillary implantation of the iris-claw lens for secondary implantations is a valid alternative strategy to the classic scleral-fixed or angle-supported IOL implantation.

## Abbreviations

RPICIOL: Retropupillary implantation of the artisan iris-claw intraocular lens
IOL: Intraocular lens
